# The Melody Valve in Small Children Undergoing First Mitral Valve Replacement: Better Than Mechanical?

**DOI:** 10.1016/j.atssr.2024.04.016

**Published:** 2024-05-06

**Authors:** Tayyaba Malik, Robert Douglas B. Jaquiss, Omar Harirah, Ryan R. Davies, Nicholas Andersen, Steven Leonard, Song Zhang, Karl M. Reyes

**Affiliations:** 1 Department of Cardiovascular and Thoracic Surgery, University of Texas Southwestern Medical Center and Children’s Medical Center, Dallas, Texas; 2 School of Public Health, University of Texas Southwestern Medical Center, Dallas, Texas

## Abstract

**Background:**

In small children with left atrioventricular valve dysfunction, valve repair is preferred but some will require valve replacement. No prosthetic valve has growth potential, bioprostheses have poor durability, and mechanical prostheses have high rates of thromboembolic and hemorrhagic complications. We reviewed our experience with a modified bovine jugular vein valve designed for use in the right ventricular outflow tract (Melody valve, Medtronic) and compared this with contemporary mechanical valve replacement.

**Methods:**

We reviewed outcomes of all left-sided atrioventricular valve replacements (AVVR) performed between January 2017 and April 2023 on patients with biventricular circulation less than 5 years old at time of surgery, N = 19. We compared mortality and major morbidity outcomes between mechanical AVVR (mAVVR, n = 14) and Melody AVVR (MAVVR, n=5).

**Results:**

MAVVR was performed on younger and smaller patients (5.7 ± 4.2 vs 28.4 ± 22.3 months, *P* = .002; 6 ± 1.7 vs10 ± 5.1 kg, *P* = .01). There were 2 early and 1 late death in the mAVVR group, and none in the MAVVR group. There was a trend towards more hemorrhagic and neurologic complications in the mAVVR group (*P* = .1, 0.28, respectively).

**Conclusions:**

In this small series, despite its use in younger and smaller patients, our preliminary experience with the Melody valve compares favorably with our contemporaneous outcomes with mechanical prostheses by way of simpler anticoagulation management with a trend towards better overall outcomes.

**In Short**

▪ Children requiring mitral valve replacement are difficult to manage with limited durable options.
▪ The Melody valve (Medtronic) in the mitral position is an option that in our small series appears equal to or not worse than mechanical valve replacement.

Mitral valve repair is preferred to mitral valve replacement (MVR) in patients of all ages, but this is particularly true in infants and children. Issues of growth potential (all valves); very poor durability (bioprostheses); too-small devices necessitating nonanatomic placement (all valves); and major difficulties with anticoagulation, both hemorrhagic and thromboembolic (mechanical prostheses), represent major drawbacks to valve replacement. Disappointing outcomes with MVR in small children are well described.^1^, ^2^, ^3^ In this context, use of Melody valves (stent-mounted bovine jugular vein valve; Medtronic) for MVR has emerged as a promising alternative,^4^ with the concept that Melody valves would function as a bridge to re-replacement with a mechanical prosthesis of larger size, and when anticoagulation would be more manageable.

Based on our disappointing results with mechanical MVR, particularly in the smallest patients, we began to implant the Melody valve. Herein we report our preliminary experience and present contemporaneous outcomes for children undergoing mechanical MVR. Note that though many of these patients had atrioventricular septal defects (AVSDs), and thus no true “mitral valve”, we have elected to use the term mitral for brevity and clarity.

## Patients and Methods

### Patients

This study was given exempt status by the institutional review board at the University of Texas Southwestern Medical Center and Children’s Medical Center. Review was performed on consecutive patients under 5 years of age with biventricular physiology who underwent atrioventricular valve replacement (AVVR) either with a mechanical valve (mAVVR) or Melody valve (MAVVR) from January 1, 2017, to April 30, 2023.

Nineteen patients were identified (14 mAVR and 5 MAVR). Statistical analysis was performed using SAS 9.4 (SAS Institute). Continuous variables were analyzed using *t* test and Wilcoxon rank-sum test, categorical variables were analyzed using Fisher's Exact test. Primary end points included risk of thromboembolic and hemorrhagic complications as well as survival and reoperation.

### Surgical Technique

**Figure 1:**
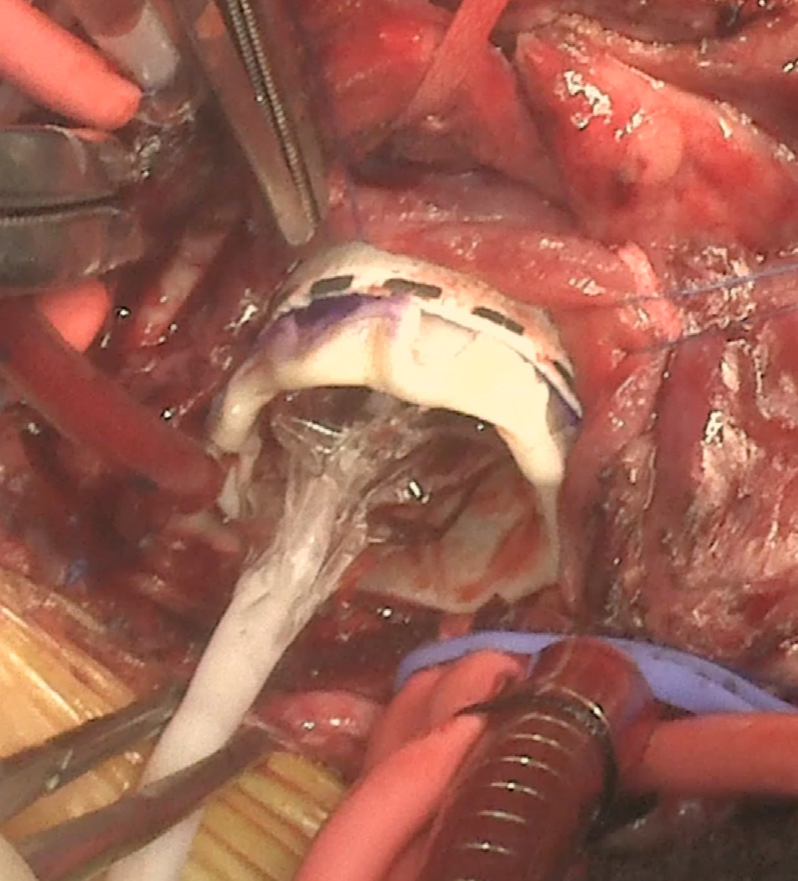
Melody valve (Medtronic) deployment into polytetrafluoroethylene graft.

Implantation at our institution is a modification of the technique by Dr James Hammel.^5^ Once valve is deemed irreparable, all atrioventricular valvar and subvalvar tissue are excised. An expandable polytetrafluoroethylene (ePTFE) tube graft (PECA exGraft) with same annular diameter as the mitral annulus is cut to 15 mm length. The graft is sutured to the mitral annulus using running polypropylene suture and knots are tied over an appropriately sized dilator to prevent a purse-string effect. The Melody valve, which has a standard total length of 30 mm, is prepared at the back table. Proximal and distal struts are folded back on itself, reducing its total length to 20 mm. The valve is then crimped and mounted on a balloon catheter. It is then deployed inside the ePTFE graft and balloon inflation is performed. During this maneuver, overdistension is avoided to prevent injury to the atrioventricular node or compression of left circumflex artery (Figure 1). The 20-mm valve in positioned in the 15-mm PTFE cylinder, keeping all stent material within the left atrium to minimize left ventricular outflow tract obstruction. Tacking sutures are then placed to prevent migration of the Melody valve. Proper positioning and valve function is confirmed with transesophageal or epicardial echocardiography (Video 1).

Postoperatively, patients are managed with aspirin and enoxaparin for 3 months and aspirin alone thereafter.

## Results

During the study period, 14 patients underwent mAVVR and 5 underwent MAVVR (Table 1).

**Table 1:** Patient Demographic

Variable	mAVVR (n = 14)	MAVVR (n = 5)	*P* Value
Age, d	865 ± 667	174 ± 124	.002
Weight, kg	10 ± 5	6 ± 2	.02
Previous mitral surgeries			
Repair	6	3	
Replacement	3	0	
Indication			
Stenosis	3	1	
Regurgitation	7	4	
Mixed mitral disease	4	0	
CPB time, min	158 ± 104	128 ± 53	.53
Cross clamp time, min	106 ± 58	61 ± 20	.02
Valve size, mm	19 ± 3	16 ± 2	.55

Values are presented as number or mean ± SD. The underline means a significant *P* value (<.05).

CPB, cardiopulmonary bypass; mAVVR, mechanical atrioventricular valve replacement; MAVVR, Melody (Medtronic) atrioventricular valve replacement.

The mean duration of clinical follow-up in the mAVVR group was 25.66 ± 11.8 months, compared with 25.8 ± 16.9 months in the MAVVR group.

For the mAVVR cohort, 7 patients had AVSD and 7 had congenitally dysplastic valves. Nine patients underwent previous mitral valve surgery, and 5 patients underwent primary mechanical valve replacement. For the MAVVR cohort, 3 patients had AVSD and 2 patients had congenitally dysplastic mitral valves. The 3 with AVSD had prior surgical repairs, the 2 dysplastic mitral valves were deemed nonrepairable. Indication for valve replacement in this group was regurgitation in 4 and stenosis in 1 patient.

Patients who underwent MAVVR were younger (mean age at the time of surgery in the mAVVR group, 5.7 ± 4.2 vs 28.4 ± 22.3 months, *P* = .002). There was no statistically significant difference in the mean cardiopulmonary bypass time (158 vs 128 minutes mAVVR and MAVVR, respectively), although the crossclamp time was significantly shorter in the MAVVR group (106 vs 61 minutes, *P* = .02). Mean size of valve implant was 19 ± 3 mm for mAVVR and 16 ± 2 mm for MAVVR, *P* = .55 (Table 1). Nominal size of the implanted Melody valve was 20 mm, but actual valve was only inflated to the size of the PTFE tube at the time of deployment.

### Early Outcomes

In the mAVVR group, 3 patients (21%) had severe left ventricular dysfunction (1 was placed on extracorporeal membrane oxygenation support postoperatively and 2 patients required permanent pacemaker placement (14%) in the immediate postoperative period, while none of these complications occurred in the MAVVR group. Trend towards more complications were present in the mAVVR group: early neurologic (seizure, stroke) (24% vs 0%, *P* = .52), respiratory (reintubation, prolonged intubation, pneumonia, acute respiratory distress syndrome) (69% vs 20%, *P* = .11), and renal complications (acute kidney injury) (31% vs 0%, *P* = .11), none of which reached statistical significance. There were 2 operative mortalities in mAVVR group and none in MAVVR group, *P* = 1.0 (Table 2).

**Table 2:** Early and Late Outcomes

Variable	mAVVR (n = 14)	MAVVR (n = 5)	*P* Value
Early			
Postoperative ECMO	1	0	1.00
Immediate postoperative complication			
CHB	2 (14)	0 (0)	1.00
LV dysfunction	3 (21)	0 (0)	.53
Days intubated	2 (1-5)	1 (1-5)	.74
ICU LOS, d	12 (6-21)	6 (5-12)	.89
Neurologic complication	3 (23.8)	0 (0)	.52
Respiratory complication	9 (69)	1 (20)	.11
Acute kidney injury	4 (31)	0 (0)	.11
Surgical mortality	2 (14)	0 (0)	1.0
Late			
Mortality at 6 mo	1 (8.3)	0 (0)	1.00
Hemorrhagic complications	7 (54)	0 (0)	.10
Thromboembolic complication			
Stroke	4 (31)	0 (0)	.28
Valve thrombosis	2 (14)	1 (20)	1.00
Readmission for anticoagulation	6 (50)	0 (0)	.10
Re-intervention within 36 mo			
Reoperation	3 (21)	1 (20)	1.00
Catheterization	6 (43)	1 (20)	.61

Values are presented as n (%) or mean (range).

CHB, complete heart block; ECMO, extracorporeal membrane oxygenation; ICU, intensive care unit; LOS, length of stay; LV, left ventricular; mAVVR, mechanical atrioventricular valve replacement; MAVVR, Melody (Medtronic) atrioventricular valve replacement.

### Late Outcomes

**Figure 2:**
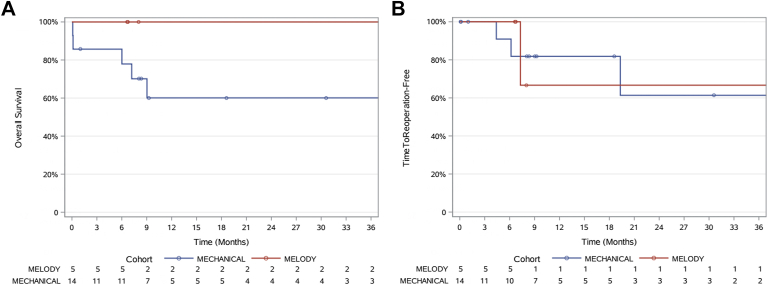
(A) Kaplan-Meier survival curve. (B) Kaplan-Meier curve for reoperation within 6 months.

Late hemorrhagic complications, including intracranial, pulmonary, and gastrointestinal bleeding, were reported in 7 mAVVR patients (54%, *P* = .1). Readmission rate for inpatient adjustment of anticoagulation was common for patients who underwent mAVVR, though not statistically different than MAVVR patients (50% vs 0, *P* = .1). One patient in the MAVVR group required early catheterization and reoperation within 36 months due to left ventricular outflow tract obstruction. Three patients who underwent mAVVR required reoperation within 36 months. These were for placement of a left ventricular assist device, left ventricular pseudoaneurysm repair, and redo replacement with Melody valve with repair of pulmonary vein stenosis. Six patients with mechanical valves had unplanned return to catheterization lab for assessment of leaflet mobility and direct thrombolytic therapy for valve thrombosis. There was 1 late mortality in the mAVVR group, and no late deaths in the MAVVR group (Figures 2A, 2B).

## Comment

Though repair of a congenitally abnormal mitral valves remains the approach of choice, this will not be successful in all patients. Options for safe and durable valve replacement are limited due to smaller mitral annular size with attendant concerns related to somatic growth and eventual patient-prosthesis mismatch, leaflet failure due to accelerated degeneration of bioprostheses, and high incidence of thromboembolic phenomena in mechanical prostheses.^6^, ^7^, ^8^ Reported experience with the Melody valve as a partial solution to this problem is certainly compatible with the experience in this present report. Despite its use in a smaller and younger cohort, and while admittedly the sample size limits any statistical inference, the Melody valve trended towards fewer complications while on a simpler patient anticoagulation regimen.^9^

In this study, there was less incidence of left ventricular dysfunction, heart block, and need for mechanical circulatory support in the MAVVR group. There were also no deaths. This may reflect the technique itself of Melody valve implantation, which appears to result in less intraoperative morbidity. This also reflects the technical challenge of implanting a mechanical prosthesis in the left atrium of young patients. Often the intent is to implant the largest possible prosthesis, which may result in compression of important intracardiac structures (conduction tissue, coronary arteries) or obstruction (pulmonary veins, left ventricular outflow tract). Other series report similar trends in morbidity^6^, ^7^, ^8^ and, to further delineate the significance of these findings, larger multicenter studies are necessary.

Intermediate term outcomes in this difficult to manage patient population may ultimately favor MAVVR as we continue to utilize this technique. Overall incidence of hemorrhagic complications defined as intracranial, pulmonary or gastrointestinal bleeding requiring readmission was lower and the overall mortality and survival favor MAVVR (Figure 2A). However, the follow-up is short, this group of patients is typically extremely ill and with multiple comorbidities, which include pulmonary hypertension and malnutrition at the time of surgery.

The durability of the Melody valve in the mitral position is short and concern for accelerated valve deterioration persists. However, potential for balloon redilation of the Melody valve as the patient grows makes it more attractive than traditional bioprostheses. Limited data exist on its longevity. Choi and associates^2^ reported the median time to redo MVR as 3.7 years for MAVVR in their institution, whereas Pluchinotta and colleagues^9^ reported a median time of 22 months. In our study, 2 patients in the MAVVR group required redo MVR after a median time of 33 months. None of the patients in our series have undergone balloon redilation to date. Regardless, the durability of the Melody valve (or any bioprosthesis) is offset by the simpler anticoagulation strategy and trend to less morbidity, which in turn results in fewer readmissions.

Criteria for choosing MAVVR instead of mAVVR is evolving and there are no set guidelines. Currently at our institution, patients younger than 5 years old with biventricular physiology are considered for MAVVR if left atrioventricular valve/mitral valve annular size is <19 mm or if they had previously undergone mAVVR but cannot tolerate anticoagulation (Figure 2B).

### Limitations

This is a single-center retrospective study with a small sample size, and the experience must be considered preliminary. The adoption of this strategy at our institution is relatively new, and hence the follow-up is short for this group.

### Conclusion

In this single institution series of infants and small children who underwent MVR, recent experience suggests that Melody valve implantation in the mitral position may be associated with less early and late mortality, fewer intraoperative complications, and avoids thromboembolic and hemorrhagic morbidity. Recent positive experience at our institution has evolved our strategy into utilizing this as the primary bridge to a durable prosthesis.
